# Temperature Dependence of Rate Processes Beyond Arrhenius and Eyring: Activation and Transitivity

**DOI:** 10.3389/fchem.2019.00380

**Published:** 2019-05-29

**Authors:** Valter H. Carvalho-Silva, Nayara D. Coutinho, Vincenzo Aquilanti

**Affiliations:** ^1^Grupo de Química Teórica e Estrutural de Anápolis, Campus de Ciências Exatas e Tecnológicas, Universidade Estadual de Goiás, Anápolis, Brazil; ^2^Dipartimento di Chimica, Biologia e Biotecnologie, Università di Perugia, Perugia, Italy; ^3^Istituto di Struttura della Materia, Consiglio Nazionale delle Ricerche, Rome, Italy

**Keywords:** transitivity plot, Aquilanti-Mundim (AM) formula, Nakamura-Takayanagi-Sato (NTS) formula, Volgel-Fulcher-Tammann (VFT) formula, viscosity and diffusion

## Abstract

Advances in the understanding of the dependence of reaction rates from temperature, as motivated from progress in experiments and theoretical tools (e. g., molecular dynamics), are needed for the modeling of extreme environmental conditions (e.g., in astrochemistry and in the chemistry of plasmas). While investigating statistical mechanics perspectives (Aquilanti et al., 2017b, 2018), the concept of transitivity was introduced as a measure for the propensity for a reaction to occur. The *Transitivity plot* is here defined as the reciprocal of the apparent activation energy vs. reciprocal absolute temperature. Since the transitivity function regulates transit in physicochemical transformations, not necessarily involving reference to transition-state hypothesis of Eyring, an extended version is here proposed to cope with general types of transformations. The transitivity plot permits a representation where deviations from Arrhenius behavior are given a geometrical meaning and make explicit a positive or negative linear dependence of transitivity for *sub*- and *super*-Arrhenius cases, respectively. To first-order in reciprocal temperature, the transitivity function models deviations from linearity in Arrhenius plots as originally proposed by Aquilanti and Mundim: when deviations are increasingly larger, other phenomenological formulas, such as Vogel-Fulcher-Tammann, Nakamura-Takayanagi-Sato, and Aquilanti-Sanches-Coutinho-Carvalho are here rediscussed from the transitivity concept perspective and with in a general context. Emphasized is the interest of introducing into this context modifications to a very successful tool of theoretical kinetics, Eyring's Transition-State Theory: considering the behavior of the transitivity function at low temperatures, in order to describe deviation from Arrhenius behavior under the quantum tunneling regime, a “*d*-TST” formulation was previously introduced (Carvalho-Silva et al., 2017). In this paper, a special attention is dedicated to a derivation of the temperature dependence of viscosity, making explicit reference to feature of the transitivity function, which in this case generally exhibits a *super*-Arrhenius behavior. This is of relevance also for advantages of using the transitivity function for diffusion-controlled phenomena.

## Introduction

To understand and control the physical chemistry of materials in an ample variety of environments that may be encountered in basic and applied scientific research, information on the kinetics of the involved elementary processes and their role in global mechanisms is needed: of particular interest are the rates, and often in a wide range of conditions—specifically of temperature. Theoretical and computational studies are of increasing utility, especially in the cases where experimental results are difficult to obtain, or the measurements are difficult to interpret. Examples span all of chemistry: from the long list that is continuously updated, we refer here to some selected cases from: combustion chemistry (Atkinson, 1986); condensed-phase (Limbach et al., 2006), atmospheric and astrochemical reactions (Smith, 2008; Sims, 2013); processes involved in preservation and aging of food and drugs (Darrington and Jiao, 2004; Peleg et al., 2012) as well as in basic geochemical (e.g., Giordano and Russell, 2018) and biochemical environments (e.g., Klinman and Kohen, 2013; Warshel and Bora, 2016).

A variety of techniques has been applied with remarkable success in several scenarios to investigate the mechanisms to both calculate and interpret the kinetics of reactive processes at a microscopic level (Sikorski et al., 2004; Pu et al., 2006; Wang et al., 2012; Hassanali et al., 2014; Coutinho et al., 2015a; Santin et al., 2016; Roy et al., 2017). From the early Arrhenius (1889) and Eyring (1935) formulations, demands emerge for interpretative theoretical tools to study the kinetics of chemical reactions and to phenomenological account of reaction rate data as generated from exact quantum benchmarks or from approximate semi-classical and classical trajectory approaches.

The seminal phenomenological description of the reaction rate constants, date of birth of theoretical chemical kinetics as a science, can be traced back to 1889 with the empirical formulation of the Arrhenius formula (Arrhenius, 1889; Laidler, 1987).

(1) $$\begin{array}{l} k=Ae^{\frac{-E_{a}}{RT}}, \end{array}$$

where *R* is the gas constant and *T* is the absolute temperature. The pre-factor *A* (often found to be temperature independent) has sometimes been given the meaning and the name of a “frequency factor.” The quantity *E*_*a*_ is termed the “energy of activation” of the reaction; according to the Arrhenius interpretation, it represents the energy that the molecule in the initial state of the process must acquire before it can take part in the reaction, whether it be a physical or a chemical process. In the Arrhenius plane prompted by Equation (1) the logarithm of a reaction rate constant, ln *k*(*T*) is plotted against reciprocal temperature, $\frac{1}{T}$: for systems that obey the Arrhenius law, Equation (1), a linear behavior is observed.

Formulations from first principles of theoretical reaction rates only became realistic after the advent of quantum and statistical mechanics. The Eyring formulation (~1935) proposes a consistent and predictive theory for the kinetic reaction rate constant: the celebrated Transition-State Theory (TST) (Eyring, 1935; Glasstone et al., 1941) provided the basis for the understanding of many phenomena and triggered most of the subsequent proposals for the understanding of physicochemical rate processes. However, as traditionally implemented, TST is unable to cope with systems with strong deviation from Arrhenius behavior (Masgrau et al., 2003). The chemical reactions for which quantum tunneling effects play an important role are those where Arrhenius plots show a concave curvature (Limbach et al., 2006; Silva et al., 2013; Sanches-Neto et al., 2017): this is the most important case of *sub*-Arrhenius kinetics for elementary reactions, but in complex processes it may show up, e.g., when concurrent reactions contribute to the mechanism (Hulett, 1964; Perlmutter-Hayman, 1976; Vyazovkin, 2016).

Eyring himself amplified the scope of his TST beyond elementary reactions proposing a formulation for including the description of viscosity and diffusion of fluids in physicochemical rate processes (Glasstone et al., 1941). However, an ample set of old and more recent data in wide ranges of temperature has been showing again and again a strong convex curvature in Arrhenius plot for both viscosity and diffusion in fluids (Angell, 1995; Coutinho et al., 2015b; Giordano and Russell, 2018). There are examples of *super*-Arrhenius kinetics, rare for elementary processes (Truhlar and Kohen, 2001), but that in complex processes are common, in particular when consecutive reactions contribute to the mechanism (Hulett, 1964; Perlmutter-Hayman, 1976; Vyazovkin, 2016): these are characteristic cases of *super*-Arrhenius kinetics for which the traditional Eyring's transition-state formulation fails. However, the TST connection between the potential energy surface profile with the phenomenological apparent activation energy through Tolman Theorem (Tolman, 1920), serves as a guide toward an interpretation of deviation from Arrhenius behavior.

In previous work (Aquilanti et al., 2017b, 2018) evidence emerged for introducing the phenomenological *Transitivity function* γ(*T*) which regulates transit in physicochemical transformations and can be put into a relationship with traditional and recent reaction rate constant formulas available in the literature. With respect to other popular phenomenological approaches, ours arguably offers flexibility for the description of the experimental data over a wide range of temperature alternative to other formulas, that were applied to various sets of problems, ranging from particle diffusion and viscosity in supercooled liquids and glasses (Angell, 2002; Stillinger and Debenedetti, 2013) to food and drug preservation and aging processes (Peleg et al., 2012).

The still popular Kooij formula (Kooij, 1893) involving an arbitrary *T*^*n*^ parameter multiplying the pre-factor *A* has no justification and is often unable to reproduce observations. Kooij formulation has to be discouraged and is physically unrealistic. It should be abandoned because: (i) at high temperature, the Arrhenius Activation energy is not recovered; (ii) at intermediate temperatures, the non-Arrhenius description is illusory valid only in extremely narrow ranges and is mathematically arbitrary; (iii) at low and ultra-low temperatures, there is consensus that non-Arrhenius behavior is pronounced, and the Kooij formula tends to Arrhenius law in clear disagreement with transitivity concept. Also, the Arrhenius-Break Temperature (ABT) formulation (Kumamoto et al., 1971; Kubo, 1985) is often one commonly used: it involves two additional parameters beyond Arrhenius and may turn out misleading from an interpretative viewpoint—when possible, it should be avoided in compacting data for modeling.

The suitability of the transitivity function is being checked against a variety of phenomenological examples with respect to its power to account for deviation from Arrhenius behavior. Here, we will show details on the treatment for amplifying the insight in various directions (section Transitivity Defined). In the subsequent section, specifically regarding the *super*-Arrhenius case, we establish the connections with the Vogel-Fulcher-Tammann treatments (VFT) (see Vogel, 1921; Fulcher, 1925; Tammann and Hesse, 1926) via the transitivity function. We also generalize the *sub*-Arrhenius case discussing in a uniform way the trend toward Wigner' limit (Wigner, 1948), yielding Nakamura-Takayanagi-Sato (NTS) formula (Nakamura et al., 1989) and Aquilanti-Sanches-Coutinho-Carvalho (ASCC) (Coutinho et al., 2018b) at low temperature. In section Transition-State Theory Extended to Moderate Tunneling (*d*-TST), the *sub*-Arrhenius case appropriate for extending the Transition-State Theory of Eyring (the *d*-TST formalism) is accounted for, as introduced and applied recently (Claudino et al., 2016; Carvalho-Silva et al., 2017; Sanches-Neto et al., 2017). A special attention will be devoted in section Viscosity and Diffusion From the Transitivity Function to a derivation of the temperature dependence of viscosity of fluids from the transitivity function γ according for the *super*-Arrhenius behavior and establishing the connection with the diffusion coefficient through the Stokes-Einstein equation. The final section is devoted to additional and concluding remarks.

## Transitivity Defined

### The Transitivity Plot

In this section, we will show how the properly defined “Transitivity” function γ(*T*) regulates the transit in physicochemical transformations: in other words, it controls the rate of passage with no bias from the transition-state hypothesis. In the 1976 article of Berta Perlmutter-Hayman (Perlmutter-Hayman, 1976) the concept of apparent activation energy *E*_*a*_ has been considered in a very deep detail: in her spirit 20 years later, the International Union of Pure and Applied Chemistry (Laidler, 1996) recommended the now accepted definition:

(2) $$\begin{array}{l} E_{a}\left(T\right)=RT^{2}\frac{d\ln k\left(T\right)}{\text{d }T}=-R\frac{\text{d}\ln k\left(T\right)}{\text{d }\left(\frac{1}{T}\right)} \end{array}$$

To be consistent with Equation (1), the assumption of constancy for *E*_*a*_ can be taken as valid for physicochemical processes, at least for the temperature range of interest but deviation occur. According to our classification of *d*-Arrhenius cases (Silva et al., 2013; Aquilanti et al., 2017a), the deviations are considered as exhibiting *sub*-, *super*-, or *anti*-types of behavior.

An initial step in order to find out how to account in a simple form for these deviations, we search for a functional dependence of *E*_*a*_ according to a large variety of cases accumulated from experiments and simulations. In her thorough study, a few decades-old, Perlmutter-Hayman (Perlmutter-Hayman, 1976) considered a large body of documentation where the dependence were regarded either as

(3) $$\begin{array}{l} E_{\text{a}}vs.T\;or\;E_{\text{a}}vs.1/T, \end{array}$$

the latter form clearly inspired by the Arrhenius plot. We have been showing ample phenomenological evidence and deep theoretical motivation (Aquilanti et al., 2017b) of the insight to be gained by studying

(4) $$\begin{array}{l} \frac{1}{E_{a}}\;vs\;\frac{1}{T}, \end{array}$$

i.e., to study the behavior of the reciprocal the activation energy studied against reciprocal of absolute temperature. Therefore, from now on, we find convenient to adopt the usual definition in statistical mechanics, of the parameter $\beta =\frac{1}{RT}$ sometime referred to as “the coldness” (e.g., Müller, 1971) and often referred as to the Lagrange parameter, because of its ubiquitous role in statistical mechanics where it occurs in optimization procedures involving the Lagrange multipliers.

From a decade-old work (Aquilanti et al., 2010), the observation arose that the reciprocal of *E*_a_ vs. the reciprocal of absolute temperature yielded an approximate linearization by the *d* parameter (italic symbol for the linearization parameter should not be confused with the roman d denoting differentials). Writing as customary the Arrhenius-Eyring energy barrier, ε^‡^, as essentially an energetic obstacle toward reaction, we have

(5) $$\begin{array}{l} \frac{1}{E_{a}}=\frac{1}{\varepsilon^{‡}}-\frac{d}{RT}\;. \end{array}$$

Introducing the “transitivity” function through the identifications

(6) $$\begin{array}{l} \gamma \left(T\right)\equiv \frac{1}{E_{a}\left(T\right)}\;, \end{array}$$

and also putting

(7) $$\begin{array}{l} \alpha =1/\varepsilon^{‡}, \end{array}$$

Equation (5) takes the simple form

(8) $$\begin{array}{l} \gamma \left(\beta \right)=\alpha -d\beta . \end{array}$$

In general, as discussed in preceding work (Aquilanti et al., 2017b), a linear γ dependence from β may be only valid in a specific range around a value β_0_; on a wider range, we can always assume that the function is well-behaved, namely that it has a Laurent power expansion,

(9) $$\begin{array}{l} \gamma \left(\beta \right)=\overset{\infty }{\underset{n=-\infty }{\sum }}c_{n}{\left(\beta -\beta_{0}\right)}^{n}. \end{array}$$

where the *c*_*n*_ coefficients are related to *n*-order derivatives of γ(β) with respect to β, taken at β_0_.

**Figure 1:**
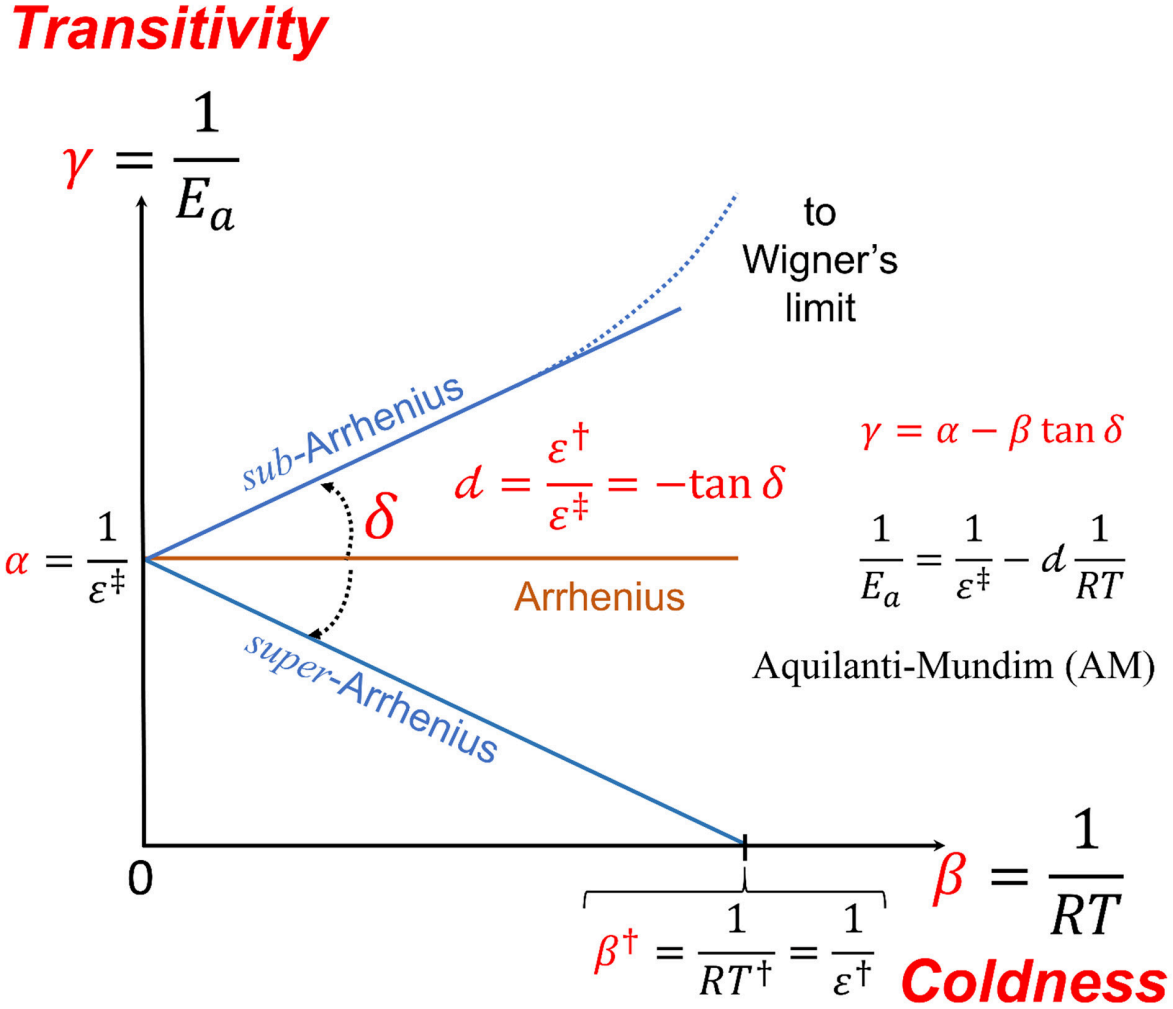
The transitivity plane, $\gamma =\frac{1}{E_{a}}\;$vs. $\beta =\frac{1}{RT}$ serves to give a geometrical meaning to the phenomenological parameters occurring in the study of non-linear Arrhenius plots. The Arrhenius behavior is given as corresponding to a line parallel to the β axis starting at $\alpha =\frac{1}{\varepsilon^{‡}}$ and corresponds to a constant apparent activation energy *E*_*a*_. The well-known double dagger notation was introduced by Eyring (Eyring, 1935; Glasstone et al., 1941). Deviations from Arrhenius behavior gives to the γ function straight line dependence at small β a direction forming the δ angle, which it is connected to the *d* parameter of the Aquilanti-Mundim (AM) law. At low temperatures as the “coldness” variable β increases, the transitivity function tends to characteristic ultra-cold limiting values: (i) for *d* < 0 (*sub*-Arrhenius) it tends to the Wigner limit and (ii) for *d* > 0 (*super*-Arrhenius), γ, namely the propensity for reaction to occur, vanishes in β^†^, γ(β^†^) = 0: the corresponding energy and temperature parameters are denoted by a single dagger, ε^†^and *T*^†^, respectively, as detailed below.

The task of a theory of the kinetics of chemical reactions is therefore focused at that of providing a set of such coefficients to connect to experimental or computationally generated *k*(*T*) via Equations (2 and 6). Advantages now are shown for the introduction of the “Transitivity Plot,” defining as the plane γ vs. β, which gives insight into the idea of the “canonical” dependence of the γ function in regulating transitions in physicochemical processes (see Figure 1).

Consistently with the established nomenclature, one gets a positive linear dependence of γ(β) for *sub*-Arrhenius (and negative for *super*-Arrhenius, *d* > 0): this according to experimental and theoretical evidence from many sources (Aquilanti et al., 2010; Silva et al., 2013). Defining in the transitivity plot $\alpha =\frac{1}{\varepsilon^{‡}}$, Equation (7), as a horizontal line (the Arrhenius line), the line of deviations from Arrhenius around β_0_ forms δ angle which can show *sub*- or *super*-Arrhenius-type of behavior, corresponding to anticlock—and clockwise direction from the β axis, respectively, yielding an expression corresponding to Equation (8),

(10) $$\begin{array}{l} \gamma =\alpha +\;\beta \tan\delta ;-tan\delta =d, \end{array}$$

where *d* < 0 (δ > 0) corresponds to the *sub*- case while *d* > 0 (δ < 0) corresponds to the *super*-Arrhenius case. Rarer cases are found for which *d* > 0 and α < 0, and are referred as corresponding to *anti*-Arrhenius behavior (e.g., Coutinho et al., 2015a).

The expression for the rate constant can be retrieved, integrating (10) from Equation (2). Introducing an integrating factor, *A* that accounts for a value for γ at the reference value, e.g., β = 0: we obtain the Aquilanti-Mundim (AM) or *d*-Arrhenius formula in the form:

(11) $$\begin{array}{l} k\left(\beta \right)=A{\left(1+\tan\delta \;\frac{\beta }{\alpha }\right)}^{-\cot\delta }, \end{array}$$

Through Equations (7, 10) and introducing the Lagrange parameter, we finally obtain the AM formula

(12) $$\begin{array}{l} k\left(T\right)=A{\left(1-d\frac{\varepsilon^{‡}}{RT}\right)}^{\frac{1}{d}}, \end{array}$$

in the usual notation (Aquilanti et al., 2010). The Arrhenius law $k\left(T\right)=A\;\exp\left(\frac{-E_{a}}{RT}\right)$ is obtained in two well-defined cases at β → 0 (high temperature limit) and at *d* → 0 (the “thermodynamic” limit) (Aquilanti et al., 2018).

### Limiting Behaviors for the Transitivity Function at Low and High Temperature

**Figure 2:**
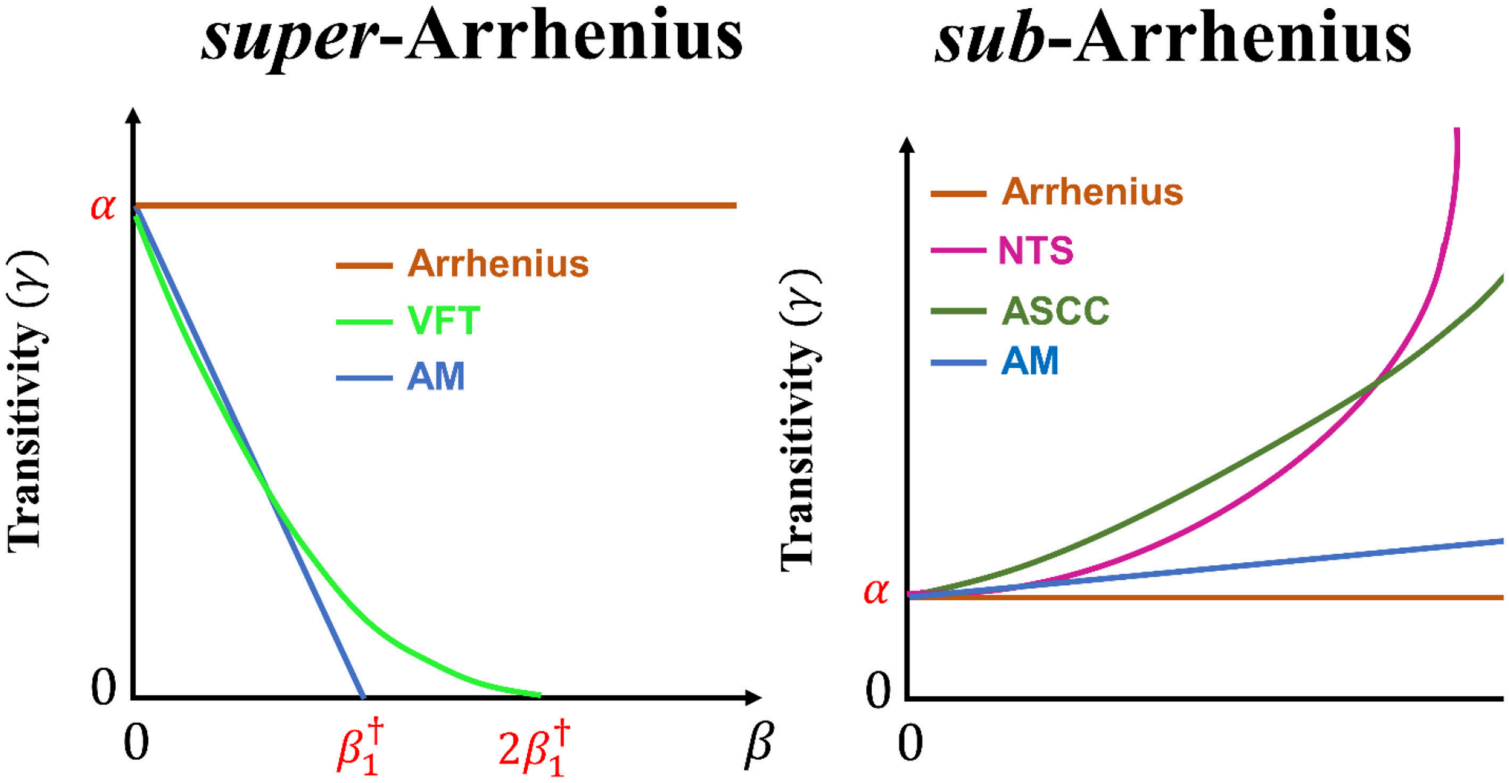
The transitivity plot is presented for venerable and recent phenomenological reaction rate constant model to account for *super*-Arrhenius behavior (Arrhenius, AM, and VFT) and *sub*-Arrhenius behavior (Arrhenius, AM, ASCC, and NTS). The comparison of the behavior of the transitivity between AM and VFT is exhibited assuming $\varepsilon_{1}^{‡}=\varepsilon_{2}^{‡}$ in Equation (26).

In the AM formulation for *k*, Equation (12), when *d* or tan δ tends to zero, one gets the exponential Arrhenius behavior through the Euler limit as detailed in Aquilanti et al. (2018). In this limit, to first order, the transitivity function deviate linearly from constancy (the Arrhenius behavior) as described by the AM formula. When β increases (low temperature):

- in the *super*-Arrhenius cases, continuing the straight-line behavior one encounters the β axis and gets for γ a zero value for a maximum attainable value of β, denoted β^†^. Here, one consequently gets a minimum value for the allowed temperature to be denoted *T*^†^. However, in generic *super*-Arrhenius behavior, sometimes high-order terms in the transitivity function are to be introduced to describe a sequence of processes, yielding concavities in the transitivity plot and moving the minimum temperature *T*^†^ at lower values (Figure 2);
- in *sub*-Arrhenius cases, the linear growth of γ as β increases may be accelerated at low temperatures: actual experimental information (Limbach et al., 2006; Tizniti et al., 2014; Meng et al., 2015) is confirmed by computations (Aquilanti et al., 2005; De Fazio et al., 2006; Cavalli et al., 2014; De Fazio, 2014; Coutinho et al., 2018b) and is eventually governed by Wigner's limit (Wigner, 1948): before the latter limit is accessed interference effects may superimpose to quantum tunneling effects, which can be studied through microcanonical exact computations (Aquilanti et al., 2005; De Fazio et al., 2006; Coutinho et al., 2018b).

We are now in the position to look at the *d*-parameter from alternative perspectives. For *super*-Arrhenius cases, the β endpoint, β^†^, marks the final low-temperature range for the system to be “active”: energetically, the introduction of the corresponding energy ε^†^ = *RT*^†^, permits the identification

(13) $$\begin{array}{l} d=\frac{\varepsilon^{†}}{\varepsilon^{‡}}. \end{array}$$

For *sub*-Arrhenius, the connection of *d* with features of the potential energy barrier permits to describe quantum tunneling in elementary chemical reactions (see e.g., Silva et al., 2013 and next section).

Additional insight to the AM formula in Equation (5) for *d* < 0 is obtained when β tends to infinity yielding,

(14) $$\begin{array}{l} \underset{\beta \to \infty }{\lim}\gamma (\beta )=-d\;\beta . \end{array}$$

Going back to the rate constant, integrating (14) from Equation (2), we obtain

(15) $$\begin{array}{l} \underset{\beta \to \infty }{\lim}k\left(\beta \right)=A\;\beta^{\frac{1}{d}}, \end{array}$$

formally appearing as the venerable Esson-Harcourt formula (Laidler, 1984), and in consonance with the Wigner limit restricted to the case of thermoneutral reactions at ultra-low temperature (Takayanagi, 2004). Nevertheless, for *d* > 0 a minimum and constant reactivity is obtained for β^†^,

(16) $$\begin{array}{l} k\left(\beta \right)=A\;{\left(\beta^{†}\right)}^{\frac{\varepsilon^{‡}}{\varepsilon^{†}}}. \end{array}$$

In Equation (16), when ε^†^ tends to infinity, *k*(β) = *A* respecting the Arrhenius limit (most other formulations do not).

After the description of the *sub*- and *super*-Arrhenius cases in the limit *d* → 0 at large β, we turn now to consider the limiting behavior as β → 0, namely at high temperature. In most cases, the generic behavior is considered to be the tendency to the Arrhenius as a limit: situations may occur where this assumption has been relaxed [important examples are protein folding (Chan and Dill, 1998; Wallace et al., 2002) and reactions in sub- or super-critical solvent (Christensen and Sehested, 1983; Lukac, 1989; Marin et al., 2003)]. We can take advantage of the following useful expansion (Abe and Okamoto, 2008; Tsallis, 2009):

(17) $$\begin{array}{l} k\left(\beta \right)=A{\left(1-d\varepsilon^{‡}\beta \right)}^{\frac{1}{d}}=Ae^{-\varepsilon^{‡}\beta }[1-\frac{1}{2}d{\varepsilon^{‡}}^{2}\beta^{2}-\frac{1}{3}d^{2}{\varepsilon^{‡}}^{3}\beta^{3}\\ \;-\frac{1}{8}{\left(2d-1\right)d}^{2}{\varepsilon^{‡}}^{4}\beta^{4}+O\left(\beta^{6}\right)], \end{array}$$

Therefore, Arrhenius behavior is recovered both as β and *d* tend to zero independently:

(18) $$\underset{\begin{array}{c} d\to 0\\ \text{const }\beta \end{array}}{\lim}k\left(d,\beta \right)=\underset{\begin{array}{c} \beta \to 0\\ \text{const }d \end{array}}{\lim}k\left(d,\beta \right)=A\;e^{-\varepsilon^{‡}\beta }.$$

## Phenomenological Models of Temperature Dependence of Reaction Rate Constants through the Transitivity Function

The previous development opens the way to the next step of our study, the examination and classification through the transitivity concept of previous phenomenological proposals, assessing relationships between them and also attempting at giving a physicochemical meaning to their empirical parameters. As a bonus, more physically motivated formulas can be generated.

From Equations (2, 6), it is possible to build up the theoretical apparatus to connect experimental or computationally generated reaction rate constants to the transitivity function and *vice*-*versa*. Below traditional and recent phenomenological reaction rate constant formulas and transitivity function are presented to deal with *sub*- and *super*-Arrhenius behavior with larger deviations than those not accounted for the AM *d*-Arrhenius formula (see Figure 2): VFT, ASCC, and NTS. The basic expression

(19) $$\begin{array}{l} k\left(\beta \right)=\exp\left(-\int_{\beta_{0}}^{\beta }\frac{\text{d}\beta }{\gamma }\right). \end{array}$$

Can be employed, where clearly, *A* = *k*(β_0_) represents the initial condition (again, the differential under the integral sign is denoted by the roman letter d to avoid confusion with *d* in italic for the deviation parameters).

### Vogel–Fulcher–Tammann (VFT) Formula

It is insightful to obtain the expression for the transitivity function for perhaps the most popular equation for modeling *super*-Arrhenius behavior, the Vogel–Fulcher–Tammann (VFT) formula (Vogel, 1921; Fulcher, 1925; Tammann and Hesse, 1926), here written as a rate constant in an Arrhenius-like fashion, but involving one additional parameter:

(20) $$\begin{array}{l} k\left(T\right)=A\exp\left(\frac{B}{T-T_{0}}\right) \end{array}$$

where *A, B, and T*_0_ are the fitting parameters: they are often designated, respectively being often denoted as the pre-factor, pseudo-activation energy and VFT-temperature, respectively. It is noteworthy, that in the polymer and food science community (Angell, 1997; Peleg et al., 2002; Coutinho et al., 2015b) Equation (20) is also known as the Williams-Landel-Ferry (WLF) equation: see (Williams et al., 1955; Dudowicz et al., 2015) where the equivalence among the respective parameters is demonstrated explicitly.

The VFT transitivity function is obtained directly from the definition through the analytical logarithmic differentiation of Equation (20) with respect to β: The result is

(21) $$\begin{array}{l} \gamma \left(\beta \right)=\frac{1}{RB}-\frac{2T_{0}}{B}\beta +\frac{RT_{0}^{2}}{B}\beta^{2}, \end{array}$$

That can be worked out in a more compact representation,

(22) $$\begin{array}{l} \gamma \left(\beta \right)=\frac{1}{RB}{\left(1-RT_{0}\beta \right)}^{2}. \end{array}$$

Here, formula (22) adds insight on the VFT parametrization for γ by a summarizing comparison with the Arrhenius and with the AM formulations for the transitivity function: the following general expression,

(23) $$\begin{array}{l} \gamma_{n}\left(\beta \right)=\frac{1}{\varepsilon_{n}^{‡}}{\left(1-d_{n}\varepsilon_{n}^{‡}\beta \right)}^{n} \end{array}$$

covers three cases for different values of *n*: (i) for *n* = 0 one recovers Arrhenius formula, (ii) for *n* = 1 the AM formula is obtained, and (iii) for *n* = 2 one gets the VFT (and WLF) formula. From Figure 1, in the transitivity plane, a geometrical interpretation can be given and leads to

(24) $$\begin{array}{l} \tan\delta =\frac{\text{d}\gamma_{n}\left(\beta \right)}{\text{d }\beta } \end{array}$$

in limiting case of β tending to zero,

(25) $$\underset{\beta \to 0}{\lim}\frac{\text{d}\gamma_{n}\left(\beta \right)}{\text{d}\beta }=\underset{\beta \to 0}{\lim}-\frac{nd_{n}{\left(1-d_{n}\varepsilon_{n}^{‡}\beta \right)}^{n}}{1-d_{n}\varepsilon_{n}^{‡}\beta }=-nd_{n}$$

and the comparison between *n* = 1 (AM) and *n* = 2 (VFT) parametrizations is shown to be

(26) $$\begin{array}{l} d_{1}={2d}_{2}\;or\;\frac{\varepsilon_{1}^{†}}{\varepsilon_{1}^{‡}}=2\frac{\varepsilon_{2}^{†}}{\varepsilon_{2}^{‡}}. \end{array}$$

See details in Figure 2, where the ASCC and NTS formulas, to be discussed next, are also considered.

### Deep Tunneling and the ASCC Formula

As reported in the earlier literature (Bell, 1980; Christov, 1997), the degree of concavity in the Arrhenius plot can be correlated with the assessment of the role of tunneling in chemical reactions: the definition of a “crossover temperature,”

(27) $$\begin{array}{l} T_{c}=\frac{\hbar \nu^{‡}}{R}, \end{array}$$

permits to conventionally establish (within some arbitrariness) the ranges of tunneling regimes for a specific imaginary frequency at the top of the barrier point, consistently denoted by a double dagger, ν^‡^: classical (*T* > 4*T*_*c*_), negligible(4*T*_*c*_ > *T* > 2*T*_*c*_), moderate (2*T*_*c*_ > *T* > *T*_*c*_) and deep (*T*_*c*_ > *T*) regimes. The ranges of tunneling regimes are indicative of the importance of quantum tunneling to affect rate constants in particular cases. From a mathematical viewpoint, the AM formulation has clear limitations in the description of the deep tunneling regime toward the Wigner limit (Wigner, 1948)

(28) $$\underset{T\to 0}{\lim}k\left(T\right)\propto T^{0}$$

Details pertinent to the present discussion can be found in a very useful reference (Takayanagi et al., 1987).

As a counterpart for *sub*-Arrhenius behavior of the *super*-Arrhenius VFT formula, it is argued that cases of deep tunneling can be dealt by introducing a modified form of the AM formula (Coutinho et al., 2018b), defined as *Aquilanti-Sanches-Coutinho-Carvalho* (ASCC) expression:

(29) $$\begin{array}{l} k\left(T\right)=A{\left(1-\frac{d\varepsilon^{‡}}{k_{B}T+h\nu^{‡}}\right)}^{\frac{1}{d}}, \end{array}$$

where $d=-\frac{1}{3}{\left(\frac{h\nu^{‡}}{2\varepsilon^{‡}}\right)}^{2}$ as reported in Silva et al. (2013) and references therein. Here, the formulation introduces the three *A*, ε^‡^ and ν^‡^ parameters and reproduces the Wigner limit at low temperature, β → ∞. The ASCC transitivity function can be worked out considering the logarithmic differentiation of Equation (29) with respect to β and the result is

(30) $$\begin{array}{l} \gamma \left(\beta \right)=\frac{1}{\varepsilon^{‡}}-\frac{d\varepsilon^{‡}-2h\nu^{‡}}{\varepsilon^{‡}}\beta +\frac{h\nu^{‡}\left(d\varepsilon^{‡}-h\nu^{‡}\right)}{\varepsilon^{‡}}\beta^{2}, \end{array}$$

or in a more compact representation,

(31) $$\begin{array}{l} \gamma (\beta )=\frac{1}{\varepsilon^{‡}}\left[1+h\nu^{‡}\beta \right]\left[1-\left(d\varepsilon^{‡}-h\nu^{‡}\right)\beta \right] \end{array}$$

For small values of *dε*^‡^, an analogous to VFT formula is recovered, see Equation (22). The ASCC formula was initially applied in Coutinho et al. (2018b) for astrochemical reactions in extremely cold environments generated by “exact” benchmark quantum dynamics. More results of applications will be given elsewhere for a variety of processes that involve deep tunneling.

### Nakamura-Takayanagi-Sato (NTS) Formula

A flexible approach to describe the deep tunneling phenomenology was proposed 30 years ago by Nakamura et al. (1989) and Sato (2005): their formula evolves smoothly behavior down to low temperature and with respect to the tendency toward the Wigner limit (Wigner, 1948):

(32) $$\begin{array}{l} k\left(T\right)=A\exp\left[-\frac{\varepsilon^{‡}}{R{\left(T^{2}+T_{0}^{2}\right)}^{\frac{1}{2}}}\right], \end{array}$$

where *A*, ε^‡^ and *T*_0_ are the parameters. Again, ε^‡^ is essentially the fitting parameter bearing connection with the barrier height along the minimum energy pathway to reaction.

Also, in this case, we can work out the Nakamura-Takayanagi-Sato transitivity function

(33) $$\begin{array}{l} \gamma \left(\beta \right)=\frac{1}{\varepsilon^{‡}}{\left[1+{\left(RT_{0}\right)}^{2}\beta^{2}\right]}^{\frac{3}{2}} \end{array}$$

## Beyond Eyring

### Transition-State Theory Extended to Moderate Tunneling (*d*-TST)

Eyring's Transition-State Theory (TST) and its variants are frequently used to compute rates of chemical reactions typically assuming a well-defined activated complex. The theory has been the object of a number of studies yielding a variety of formulations based on the concept of an equilibrium between the reactants and the activated complex, all assumed with Boltzmann distributions of the internal degrees of freedom. The rate of transformation is, then, obtained by a combining of thermodynamics, kinetics, quantum chemistry, and statistical mechanics arguments. The authoritative textbook is (Glasstone et al., 1941). For a general bimolecular reaction, such as $R_{1}+R_{2}\to TS^{‡}\to Products$, it is necessary to compute the *Q*_1_, *Q*_2_, and *Q*^‡^ partition functions of *R*_1_, *R*_2_ and of the transition state, respectively. However, the conventional TST is not able to account for low temperature curvatures in the Arrhenius plot, particularly when due to quantum tunneling through the reaction barriers (for the viscosity of fluids see next section). To account for the quantum tunneling in chemical reactions, the transitivity function is modeled in analogy with the AM formula, yielding the *deformed-*Transition-State Theory (*d*-TST) (Carvalho-Silva et al., 2017):

(34) $$\begin{array}{l} k\left(T\right)=\frac{RT}{h}\frac{Q^{‡}}{Q_{1}Q_{2}}{\left(1-d\frac{\varepsilon^{‡}}{RT}\right)}^{\frac{1}{d}},\;d=-\frac{1}{3}{\left(\frac{h\nu^{‡}}{2\varepsilon^{‡}}\right)}^{2}, \end{array}$$

where *h* is the Planck's constant and ε^‡^ is the effective height of the energy barrier, given by the sum of the harmonic zero-point energy correction and the height of the potential energy barrier. This formulation uniformly covers the range from classical to moderate tunneling regimes but is inadequate for deep tunneling. The proposed variant of transition-state theory permits comparison with experiments and tests against alternative formulations (see e.g., Claudino et al., 2016; Santin et al., 2016; Sanches-Neto et al., 2017).

### Viscosity and Diffusion From the Transitivity Function

Eyring's proposal of a kinetic rate theory was also amplified toward the description of viscosity and diffusion of fluids in physicochemical processes (Eyring, 1936; Glasstone et al., 1941). Eventually, it turned out that the theory was unable to describe processes in a wide temperature range, in particular when presenting a convex curvature in the Arrhenius plot. In the present context, this is a manifestation of *super*-Arrhenius kinetics (Truhlar and Kohen, 2001; Coutinho et al., 2015b; Giordano and Russell, 2018). To describe deviations from Arrhenius of the rates of reaction in fluids, we take into account later developments by Kramers (1940) and Collins and Kimball (1949), involving viscosity and diffusion.

To account for the temperature dependence of viscosity in cases clearly exhibiting *super*-Arrhenius behavior, we introduce a treatment using the transitivity function concept. From the defining, Equations (2) and (6) we obtain the differential equation,

(35) $$\begin{array}{l} \frac{\text{d}}{\text{d }\beta }k\left(\;\beta \right)\;-\;\frac{1}{\gamma \left(\;\beta \right)}\;k\left(\;\beta \right)\;=\;0. \end{array}$$

For β_0_ = 0 as the lower limit of integration range and the restriction to only two terms of the Taylor–McLaurin series of Equation (9), we obtain the AM transitivity function, where α < 0 represents an energetic propensity toward to evolution of the fluid. The *d* is again the deformation parameter, playing an analogous role to that amply discussed previously: the result is an AM-like formula for viscosity (Aquilanti et al., 2017b),

(36) $$\begin{array}{l} \eta \;\left(\;\beta \right)\;=\;\eta_{o}\;{\left(\;1+\;d\varepsilon^{‡}\beta \right)}^{\frac{1}{d}}, \end{array}$$

here η_*o*_ is introduced as a counterpart of Arrhenius pre-factor *A* and is the viscosity when the temperature tends to infinity (β → 0). At low temperature, in viscous processes the apparent activation energy turns out to increase indefinitely and consequently the propensity to proceed to a kinetic transition approaches zero, γ → 0: so we establish a direct relationship for the *d* parameter (analogous to the cases dealt in section Limiting Behaviors for the Transitivity Function at Low and High Temperature):

(37) $$\begin{array}{l} d=\frac{RT^{†}}{\varepsilon^{‡}}, \end{array}$$

and *T*^†^ is identified as a phenomenological “freezing” temperature of the process, namely the critical temperature (to be connected with that of glass transition, see Aquilanti et al., 2017b), where the kinetic energy of the fluid particles is too low for the process to be turned on. In the early approach by Eyring (1936) and Glasstone et al. (1941), it was argued that the ε^‡^ parameter be empirically put into relationship with the energy of vaporization of the fluid, Δ*H*_*vap*_, and intuitively connected with the work required to make a hole of molecular size.

Using the Kauzmann-Eyring pre-factor $\eta_{0}=\frac{N_{a}h}{\bar{V}}$ (Kauzmann and Eyring, 1940; Glasstone et al., 1941), where *N*_*a*_ is the Avogadro number and $\bar{V}$ is the molar volume, and Equation (37) for *d*, we finally obtain,

(38) $$\begin{array}{l} \eta \left(T\right)=\frac{N_{a}h}{\bar{V}}{\left(1+\frac{T^{†}}{T}\right)}^{\frac{\varepsilon^{‡}}{RT^{†}}}. \end{array}$$

when *T*^†^ tends to zero, the Arrhenius-Eyring exponential formula for viscosity is recovered, $\eta \left(T\right)=\frac{N_{a}h}{\bar{V}}\exp\left(\frac{\varepsilon^{‡}}{RT^{†}}\right)$ through the Euler limit.

The deviation from Arrhenius behavior in the temperature dependence of diffusion can now be evaluated from Equation (39) through the Stokes-Einstein equation(Einstein, 1905).

(39) $$\begin{array}{l} D\left(T\right)=\frac{k_{B}T}{6\pi r}\frac{1}{\eta \left(T\right)} \end{array}$$

where *r* is the hydrodynamic radius (Henriksen and Hansen, 2008). This treatment of course does not provide further insight into these amply investigated issues, but points at a simple and perhaps useful physical interpretation of a long-standing as well recent intriguing rate phenomena. From a general perspective, the theory encourages considering wide ranges of available data on geochemistry (Giordano and Russell, 2018), supercooled liquids science (Angell, 1995) and biochemistry (Kohen et al., 1999) and digging for hidden insights. Preliminary searches, to be published, turned on successful.

## Additional and Final Remarks

This paper applies thoroughly the transitivity concept to a set of topics, completing the presentation of the theory outlined in Aquilanti et al. (2017b). A separated paper (Machado et al., submitted) presents the code developed for the implementation to a set of cases of interest in physicochemical kinetics where the need for deviation from Arrhenius behavior is demanded: applications of our formulation can be accessed in the homepage of our computational code—Transitivity (www.vhcsgroup.com/transitivity), where manual, installation video, and specific examples are provided. Further remarks follow:

### *Ab initio* “Exact” Quantum Dynamics

In principle, this is the most valuable source of kinetics data but still limited to simple benchmark cases. For full formulations of the reaction kinetics, following the microcanonical path along a quantum chemically or empirically generated potential energy surface, high-level computational effort is demanded. It typically proceeds according to these steps: a) calculation of the intermolecular interactions involved in a reactive process with a high-level of accuracy, b) dynamical evolution in phase-space configurations from the solution of quantum equations of motion, c) identification of reactive trajectories, with consequent calculation of the quantum scattering matrix, cumulative reaction probability and cross sections. Finally, the Boltzmann weight averaging over a large span of kinetics energies yields the canonical expressions of kinetic variables as a function of temperature. These severe prescriptions have been able to provide the exact calculation over a given potential energy surface for reaction rate constants of only a limited number of reactive systems: in fact, the complexity of the programming and the computationally demanding requirements and computational demand strongly limit the study of reactive processes involving only a few atoms. Additional reactions involving isotopic exchange among three hydrogen atoms, exemplary benchmarks to be cited are the three-body reactions: F + H_2_ (Aquilanti et al., 2005), F + HD (De Fazio et al., 2006; Cavalli et al., 2014), H + HeH^+^ (De Fazio, 2014) see also and references therein.

### First-Principles Molecular Dynamics

Another viable path is becoming possible thanks to improvements in computational facilities, in order to access at kinetic information through first-principles molecular dynamics simulations. However, computationally severe storing and time constraints permitting to obtain myriads of “on-the-fly trajectories” require a great effort toward the aim of generating realistic reactive kinetic data: this in spite of the fact that a wide research activity has been pursued, aiming at developing techniques capable of accurately predicting kinetic reaction rate constants from molecular dynamics simulations. Among examples that have been tackled in recent years, we cite (Pomerantz et al., 2005; Coutinho et al., 2015a; Döntgen et al., 2015; Fleming et al., 2016; Wu et al., 2019) and references therein. However, calculation of reaction rate constants has been limited by the arduous procedures both to accurately characterize reactive activated complexes of many body systems and to overcome the inherent difficulties of producing a number of trajectories with statistical consistency and reasonable completeness in the filling of the dynamically relevant parts of the phase-space. Recently, several works have been yielding values with reliable accuracy: overestimates due to limited sampling of phase-space, when experimental values are available for comparison, may exploit empirical calibration (Coutinho et al., 2016, 2017, 2018a).

### Phenomenological Considerations and the Role of the Transitivity Concept

Experimentally and computationally generated kinetic data for polyatomic molecules provide reaction rate constants with the implicit fingerprint of the microscopical variables at work in the reactive process (Angell, 1995; Kohen et al., 1999; Limbach et al., 2006; Giordano and Russell, 2018; Capitelli and Pietanza, 2019, and references therein). Application of the techniques discussed in previous remarks is tremendously laborious for many-body systems: when the Arrhenius law is not obeyed at low temperature transitivity function guides us to an as a fruitful and consistent approach. As discussed in this article the approach turns out to be a powerful tool, capable of establishing a connection between canonical data and microcanonical information, permitting comparisons among apparently uncorrelated formulations: it also allows interpretation of empirical parameters, for example for the AM, ASCC, VFT and NTS formulas considered in this paper. Previous (Tsallis and Bukman, 1996; Lenzi et al., 2001; JiangLin et al., 2006; Zhou and Du, 2013) and concomitant (Zhou and Du, 2014; Guo and Du, 2015; Rosa et al., 2016; Junior et al., 2019) efforts have been dedicated to provide also a connection of anomalous kinetic diffusion effects while surmounting a potential barrier *via* variants of Fokker-Planck equation, tackling a class of phenomenologically physicochemical diffusion process.

### Conclusion and Perspectives

In order to extend the validity of the Arrhenius rate law, the introduction of the deformation parameter *d* not only phenomenologically mimics the low temperature dependence of rate constants, but its relevance in producing physical insight is now amply demonstrated. The statistical mechanics aspects are now firmly established (Aquilanti et al., 2017b, 2018) capitalizing on various investigations inspired openly or implicitly on a Maxwellian approach: several examples in the literature have been inspiring the transfer from thermodynamics to the field of kinetics assuming procedures for taking the “thermodynamic limit.” Venerable papers are (Jeans, 1913; Condon, 1938; Kennard, 1938; Landau and Lifshitz, 1958), and recent ones (Tsallis, 1999; Biró et al., 2014; Aquilanti et al., 2017b).

## Data Availability

The raw data supporting the conclusions of this manuscript will be made available by the authors, without undue reservation, to any qualified researcher.

## Author Contributions

All authors listed have made a substantial, direct and intellectual contribution to the work, and approved it for publication.

### Conflict of Interest Statement

The authors declare that the research was conducted in the absence of any commercial or financial relationships that could be construed as a potential conflict of interest.
